# Intracellular oxygen tension limits muscle contraction‐induced change in muscle oxygen consumption under hypoxic conditions during Hb‐free perfusion

**DOI:** 10.14814/phy2.13112

**Published:** 2017-01-20

**Authors:** Hisashi Takakura, Minoru Ojino, Thomas Jue, Tatsuya Yamada, Yasuro Furuichi, Takeshi Hashimoto, Satoshi Iwase, Kazumi Masuda

**Affiliations:** ^1^ Faculty of Health and Sports ScienceDoshisha UniversityKyotanabeJapan; ^2^ Faculty of Human SciencesKanazawa UniversityKanazawaJapan; ^3^ Department of Biochemistry and Molecular MedicineUniversity of California DavisDavisCalifornia; ^4^ Department of Cell BiologySchool of MedicineJohns Hopkins UniversityBaltimoreMaryland; ^5^ Department of Health Promotion ScienceTokyo Metropolitan UniversityHachiojiJapan; ^6^ Faculty of Sport and Health ScienceRitsumeikan UniversityKusatsuJapan; ^7^ Department of PhysiologyAichi Medical UniversityNagakuteJapan

**Keywords:** Hindlimb perfusion, hypoxia, intracellular oxygen tension, myoglobin

## Abstract

Under acute hypoxic conditions, the muscle oxygen uptake (m$\dot{V}$O_2_) during exercise is reduced by the restriction in oxygen‐supplied volume to the mitochondria within the peripheral tissue. This suggests the existence of a factor restricting the m$\dot{V}$O_2_ under hypoxic conditions at the peripheral tissue level. Therefore, this study set out to test the hypothesis that the restriction in m$\dot{V}$O_2_ is regulated by the net decrease in intracellular oxygen tension equilibrated with myoglobin oxygen saturation (∆P_mb_O_2_) during muscle contraction under hypoxic conditions. The hindlimb of male Wistar rats (8 weeks old, *n* = 5) was perfused with hemoglobin‐free Krebs–Henseleit buffer equilibrated with three different fractions of O_2_ gas: 95.0%O_2_, 71.3%O_2_, and 47.5%O_2_. The deoxygenated myoglobin (Mb) kinetics during muscle contraction were measured under each oxygen condition with a near‐infrared spectroscopy. The ∆[deoxy‐Mb] kinetics were converted to oxygen saturation of myoglobin (S_mb_O_2_), and the P_mb_O_2_ was then calculated based on the S_mb_O_2_ and the O_2_ dissociation curve of the Mb. The S_mb_O_2_ and P_mb_O_2_ at rest decreased with the decrease in O_2_ supply, and the muscle contraction caused a further decrease in S_mb_O_2_ and P_mb_O_2_ under all O_2_ conditions. The net increase in m$\dot{V}$O_2_ from the muscle contraction (∆m$\dot{V}$O_2_) gradually decreased as the ∆P_mb_O_2_ decreased during muscle contraction. The results of this study suggest that ΔP_mb_O_2_ is a key determinant of the Δm$\dot{V}$O_2_.

## Introduction

Under acute hypoxic conditions, the decrease in O_2_ supply reduces muscle oxygen consumption (m$\dot{V}$O_2_) during exercise (Calbet et al. 2003; Lundby et al. 2004; Calbet et al. 2009). However, a series of O_2_ transport steps (ventilation, diffusion from the lung to the blood, bulk delivery by the cardiovascular system, and the transfer of O_2_ from the blood to the skeletal muscle) control the m$\dot{V}$O_2_ and oxidative ATP generation (Bassett and Howley 2000; Howlett et al. 2009). The way the various steps involved in the determination of the $\dot{V}$O_2_ contribute to the respiration during exercise under hypoxia conditions remains unclear. Recent research has reported that in hypoxia $\dot{V}$O_2_ might be limited by factors aside from arterial oxygen content (Lundby et al. 2004). Taken together, these facts suggest the existence of factors regulating the m$\dot{V}$O_2_ at the peripheral tissue level.

As the value of the m$\dot{V}$O_2_ depends on the O_2_ flux – that is, the O_2_ diffusion conductance (DO_2_) and the O_2_ gradient between the microvasculature and the myocytes – the decrease in m$\dot{V}$O_2_ under hypoxic conditions may be caused by decreases in both the DO_2_ and O_2_ gradients across the plasma membrane (Richardson et al. 1995; Takakura et al. 2010). However, previous studies have not considered the contribution of the O_2_ gradient as based on the intracellular oxygen tension equilibrated with myoglobin oxygen saturation (P_mb_O_2_) (Gonzalez et al. 2006). Currently, only the P_cap_O_2_ is regarded as an adequate measure of the O_2_ diffusion between the plasma membrane. The study assumed that the P_mb_O_2_ at rest was close to zero and that it remained unchanged during moderate‐ to high‐intensity exercise (Richardson et al. 1995). However, under hypoxic condition, Richardson et al. (2006) could not detect any deoxy myoglobin (Mb) signal at rest due to presumably a low signal to noise ratio. Therefore, it remains unclear whether muscle contraction under hypoxic conditions causes a further decrease in P_mb_O_2_. Other groups have reported that the P_mb_O_2_ decreases with the increase in exercise intensity under normoxic conditions, and that the O_2_ gradient from the vasculature to the cell could play a key role in the control of the $\dot{V}$O_2_ (Molé et al. 1999; Chung et al. 2005). These conflicting results necessitate an examination of the relationship between the m$\dot{V}$O_2_ and the intracellular O_2_ environment during muscle contraction under hypoxic conditions.

We recently demonstrated that the intracellular Mb dynamics during muscle contraction involved in enhancing the O_2_ flux to meet the increased muscle O_2_ demand (Masuda et al. 2010; Takakura et al. 2010). Indeed, the P_mb_O_2_ decreased with increasing exercise intensity under normoxic conditions (Molé et al. 1999; Takakura et al. 2010). This change in the O_2_ gradient as reflected by the decrease in the P_mb_O_2_ can contribute to the muscle O_2_ uptake. Therefore, this study considers the effect of hypoxia on the intracellular O_2_ environment and assesses whether the vasculature‐to‐cell O_2_ gradient contributes in regulating the m$\dot{V}$O_2_ during muscle contraction.

## Materials and Methods

### Experimental animals and preparation of hindlimb perfusion

Male Wistar rats were employed as subjects. All were housed in a temperature‐controlled room at 23 ± 2°C with a 12‐h light–dark cycle and maintained on a commercial diet with water ad libitum. The procedures conformed to the “Fundamental Guidelines for Proper Conduct of Animal Experiment and Related Activities in Academic Research Institutions” (published by the Ministry of Education, Culture, Sports, Science and Technology, Japan) and was approved by the Ethics Committee for Animal Experimentation of Kanazawa University (Protocol AP‐101821).

The hindlimb perfusion was performed to the rats at 8 week of age (body weight at experiment; 256.0 ± 8.7 g). Preparation of isolated rat hindlimb and the perfusion apparatus are described in previous reports (Masuda et al. 2010; Takakura et al. 2010, 2015). All surgical procedures were performed under pentobarbital sodium anesthesia (64.8 mg/kg intraperitoneal). After finishing a surgical procedure, the rats were killed by injecting 1 mol/L KCl solution directly into the heart and a hemoglobin‐free Krebs–Henseleit buffer (NaCl, 118 mmol/L; KCl, 5.9 mmol/L; KH_2_PO_4_, 1.2 mmol/L; MgSO_4_, 1.2 mmol/L; CaCl_2_, 1.8 mmol/L; NaHCO_3_, 20 mmol/L; Glucose, 15 mmol/L) equilibrated with 95% O_2_ + 5% CO_2_ at 37°C was perfused into the abdominal aorta in flow through mode, at a constant flow rate. In order to adjust the perfusion pressure to approximately 80.0 mmHg, the flow rate was set to 22.2 ± 0.5 mL min^−1^ throughout the perfusion period. In this condition, the average perfusion pressures were 77.5 ± 3.1 mmHg, and the perfusion resistance was unchanged throughout the perfusion period. In addition, no sign of edema in the hindlimb was seen at the given flow rate. The effluent was collected from the inferior vena cava in order to measure m$\dot{V}$O_2_ and the lactate and pyruvate concentrations.

During the perfusion period, the oxygen supply was modulated by adjusting the O_2_ fraction of the equilibration gas using nitrogen gas, while 5% CO_2_ concentration was maintain to keep pH in perfusate. This study set three levels of O_2_ concentration: a 95.0% O_2_ fraction, a 71.3% O_2_ fraction, and a 47.5% O_2_ fraction. After establishing a sufficient equilibrium in each oxygen condition, muscle contraction was performed on a rat at the maximal twitch tension in all oxygen conditions. The order of the three oxygen conditions was randomized.

### Measurement parameters

The twitch contraction protocol and measurement of Mb oxygenation and m$\dot{V}$O_2_ followed the previous methods (Masuda et al. 2010; Takakura et al. 2010). The sciatic nerve of the left hindlimb was then exposed and connected to two parallel stainless steel wire electrodes (Unique Medical, Tokyo, Japan) and the Achilles’ tendon was connected to a sensitive strain gauge with a string (MLT500/D, AD Instrument, Castle Hill, NSW, Australia). The stimulation pulse via the sciatic nerve derived by an electrostimulator system (Model RU‐72, Nihon Koden, Tokyo, Japan) was 1 Hz in frequency (delay, 10 *μ*sec; duration, 1 msec) for 120 sec (120 twitch contractions). Target tension was controlled by changing the voltage of stimuli to obtain 100% of peak tension under buffer‐perfused conditions (3–8 volts). Twitch tension was calculated as the average of a series of contractions. The muscle also showed no sign of fatigue at the set stimulation intensity.

An NIRS instrument (NIRO‐300 + Detection Fibre Adapter Kit, Hamamatsu Photonics, Shizuoka, Japan) was employed to measure oxygenation of Mb at rest and during muscle contraction. The distance between the photodiode and the LED was fixed at 10 mm. The toe of the foot was secured by a clamp with the rat laid on its back. After that, the NIRS probes were firmly attached to the skin of the gastrocnemius muscle and were fixed by clamps on both sides of the muscle. During the initial period, for at least 30 sec before the start of contraction, the average fluctuation in the NIRS signals was adjusted to a reference value of zero. After the exercise protocol, the anoxic buffer (equilibrated with 95% N_2_ + 5% CO_2_ gas) was perfused for 30 min to obtain maximal Mb desaturation. The muscle then received electrical stimulation to contract for 2 min. No further increase in change in NIRS signal associated with concentration of deoxygenated Mb (∆[deoxy‐Mb]) signal was evident. The final ∆[deoxy‐Mb] signal intensity served as the normalization constant for 100% Mb deoxygenation.

The value of m$\dot{V}$O_2_ was calculated from the arteriovenous oxygen content difference multiplied by the flow rate, using the equation:

$$\begin{array}{cc} m\dot{V}O_{2}\left(\mu \text{mol}\;\;g^{-1}{\text{min}}^{-1}\right)= & \;\text{flow rate}\times [\left({\text{PO}}_{2_{\mathrm{in}}}-{\text{PO}}_{2_{\mathrm{out}}}\right)\\ & \times O_{2}\;\;\text{solubility}]/\text{muscle weight} \end{array}$$

where flow rate is the flow in milliliters per minute, and PO_2in_ and PO_2out_ are the arterial and venous oxygen tensions after adjusting for the vapor pressure of water. Inflow PO_2_ and outflow PO_2_ were measured continuously using two O_2_ electrodes (5300A, YSI, Yellow Springs, OH) along tubing before and after perfusion of the hindlimb. The vapor pressure at 37°C was 47.03 mmHg. The solubility of oxygen in the buffer was 0.00135 *μ*mol mL^−1^ mmHg^−1^ at 37°C (Philip and Dorothy 1971). The m$\dot{V}$O_2_ at rest and during muscle contraction was calculated by using the values of PO_2in_−PO_2out_ averaged over 15 sec in the steady‐state conditions before and during muscle contraction.

The sampling rate for the NIRS data was 1 Hz. The other parameters (tension, perfusion pressure, O_2_ content at the inflow and outflow) were collected using a data acquisition system (PowerLab 8SP, AD Instruments, Australia) at a sampling rate of 1 kHz. All the data were transferred to a personal computer with acquisition software (Chart ver. 5.5.6. AD Instruments).

### Data analysis

The data analysis followed our previous methods (Takakura et al. 2010, 2015). A simple moving average smoothed the ∆[deoxy‐Mb] NIRS signals using a rolling average of 5 points, which corresponds to a 5 sec timeframe(Box et al. 1978). The ∆[deoxy‐Mb] signals were calibrated against two different NIRS signal values: one at rest as 10% Mb deoxygenation and the other during steady state with anoxic buffer perfusion as 100% Mb deoxygenation. While the S_mb_O_2_ at rest could not be determined by NIRS, the value was assumed to be 90% based on previous studies reporting that the S_mb_O_2_ at rest was greater than 90% (Chung et al. 2005). The %∆[deoxy‐Mb] plots were converted to S_mb_O_2_ (%) plots using the following equation:

$$S_{\mathrm{mb}}O_{2}=100-\%\Delta \left[\text{deoxy}-\text{Mb}\right]$$

S_mb_O_2_ plots were fitted by the following single‐exponential equation to calculate kinetics parameters using an iterative least‐squares technique by means of a commercial graphing/analysis package (KaleidaGraph 3.6.1, Synergy Software, Reading, PA):

$$S_{\mathrm{mb}}O_{2}=\text{BL}+\text{AP}\times \left[1-\exp^{-(t-\mathrm{TD})/\tau }\right]$$

where BL is the baseline value, AP the amplitude between BL and the steady‐state value during the exponential component, TD the time delay between onset of contraction and appearance of S_mb_O_2_ signals, and *τ* the time constant of S_mb_O_2_ signal kinetics. MRT calculated by TD + *τ* was used as an effective parameter of the response time for Mb deoxygenation at onset of muscle contraction. Dividing 63% of AP by MRT yields a value for the time‐dependent change in Mb deoxygenation. The P_mb_O_2_ value (mmHg) at rest and steady state during muscle contraction was converted from the S_mb_O_2_ value using the following equation:

$$P_{\mathrm{mb}}O_{2}=\frac{S_{\mathrm{mb}}O_{2}\cdot P_{50}}{\left(1-S_{\mathrm{mb}}O_{2}\right)}$$

where P_50_ is the partial oxygen pressure required to half‐saturate Mb. A P_50_ of 2.4 mmHg was used for this equation, assuming a muscle temperature of 37°C (Schenkman et al. 1997). The calculated P_mb_O_2_ plots were evaluated to obtain an MRT of its kinetics using the same single exponential equation as for P_mb_O_2_. The _0.63_AP/MRT for P_mb_O_2_ indicates a rate of decrease in P_mb_O_2_ at muscle contraction onset. P_mb_O_2_ at steady state was calculated by using the S_mb_O_2_ value at steady state. Since O_2_ partial pressure corresponds to a specific amount of dissolved O_2_, intracellular [O_2_] (*μ*M) was calculated from the P_mb_O_2_ value at rest and at each O_2_ condition using the following equation:

$$\text{Intracellular}\;{\left[O\right]}_{2}=P_{\mathrm{mb}}O_{2}\times O_{2}\;\text{solubility}$$

with P_mb_O_2_ is in mmHg, and O_2_ solubility in buffer is 0.00135 *μ*mol mL^−1^ mmHg^−1^ at 37°C (Philip and Dorothy 1971).

The relationship of m$\dot{V}$O_2_ to conductance and O_2_ gradient used the following equation:

$$m\dot{V}O_{2}={\text{kDO}}_{2}\times \left(P_{\mathrm{cap}}O_{2}-P_{\mathrm{mb}}O_{2}\right)$$

where m$\dot{V}$O_2_ is muscle oxygen consumption, k is constant, D is conductance, P_cap_O_2_ is microvascular oxygen tension, P_mb_O_2_ is intracellular oxygen tension equilibrated with myoglobin oxygen saturation as determined from the Mb signal. Because study used a constant flow perfused hindquarter model, the analysis has assumed a proportional relationship between the outflow and capillary PO_2_ and has set 30 mmHg as the reference the normoxic P_cap_O_2_ value (Behnke et al. 2001; McCullough et al. 2011; Kano et al. 2014; Ferguson et al. 2015).

### Statistical analyses

All data are expressed as mean ± SD. Statistical differences were examined using one‐way paired measures analysis of variance (ANOVA) (factor: O_2_ fraction). A Turkey–Kramer post hoc test was applied if the ANOVA indicated a significant difference. The level of significance was set at *P* < 0.05.

## Results

In this study, the oxygen supply to the hindlimb muscle decreased from 17.1 ± 1.3 *μ*mol min^−1^ at 95% O_2_ saturation to 12.0 ± 1.2 and 8.8 ± 0.9 *μ*mol min^−1^ in the 71.3% and 47.5% O_2_ saturation, respectively. The correspondence between the O_2_ saturation and the measured O_2_ concentration in the perfusate confirmed the appropriateness of the experimental conditions. As the O_2_ supply volume decreased, the m$\dot{V}$O_2_ in resting muscle decreased gradually (Table 1). Even though S_mb_O_2,_ and P_mb_O_2_ also decrease, the measurements could not detect a significant change in the estimated O_2_ gradient as reflected in (P_cap_O_2_‐P_mb_O_2_), and did not reveal any significant alteration. Under all hypoxia conditions, the lactate to pyruvate ratio (L/P) showed no increase (Table 1).

**Table 1:** Muscle oxygen consumption and intracellular O_2_ parameter at rest during hindlimb perfusion with different O_2_ fraction

Parameter	Unit	O_2_ fraction in pefusate		
		47.5%	71.3%	95.0%
Inflow PO_2_	mmHg	291.1 ± 37.4^a^, ^b^	399.1 ± 48.0^a^	566.9 ± 53.7
[O_2_] in perfusate	*μ*mol/L	18.0 ± 2.1^a^, ^b^	24.5 ± 2.7^a^	35.1 ± 3.0
Outflow PO_2_	mmHg	112.3 ± 20.7^a^, ^b^	170.5 ± 31.8^a^	291.1 ± 37.4
Relative outflow PO_2_		0.41 ± 0.08^a^, ^b^	0.63 ± 0.12^a^	1.00 ± 0.15
Estimated P_cap_O_2_	mmHg	12.4 ± 2.3^a^, ^b^	18.8 ± 3.5^a^	30.0 ± 4.6
m$\dot{V}$O_2_	*μ*mol g^−1^ min^−1^	0.34 ± 0.05^a^, ^b^	0.43 ± 0.07^a^	0.55 ± 0.12
S_mb_O_2_	%	55.0 ± 7.9^a^, ^b^	74.1 ± 11.7^a^	90.0 ± 0.1
P_mb_O_2_	mmHg	3.1 ± 0.9^a^, ^b^	8.5 ± 4.6^a^	21.7 ± 0.2
Intracellular [O_2_]	*μ*mol/L	4.1 ± 1.2^a^, ^b^	11.5 ± 6.2^a^	29.3 ± 0.3
P_cap_O_2_‐P_mb_O_2_	mmHg	9.3 ± 3.0	10.3 ± 5.2	8.3 ± 4.5
L/P		19.6 ± 3.0	17.8 ± 2.3	18.3 ± 2.0

Values are mean ± SD (*n* = 5 in each condition). Inflow PO_2_, oxygen tension before perfusion of the hindlimb; [O_2_] in perfusate, O_2_ concentration in perfusate; Outflow PO_2_, oxygen tension after perfusion of the hindlimb; Relative outflow PO_2_, relative value of outflow PO_2_ based on the outflow PO_2_ value at 95.0%O_2_ fraction; Estimated P_cap_O_2_, estimated microvasucular oxygen tension based on 30 mmHg of P_cap_O_2_ at 95.0%O_2_ fraction. m$\dot{V}$O_2_, muscle oxygen consumption; S_mb_O_2_, intracellular O_2_ myoglobin saturation; P_mb_O_2_: intracellular O_2_ tension equilibrated with myoglobin O_2_ saturation; Intracellular [O_2_], intracellular O_2_ concentration; P_cap_O_2_‐P_mb_O_2_, the difference in oxygen tension between P_cap_O_2_‐P_mb_O_2_; L/P, lactate to pyruvate ratio measured in effluent perfusate.

a *P* < 0.05 versus 95.0% condition

b *P* < 0.05 versus 71.3% condition.

**Figure 1:**
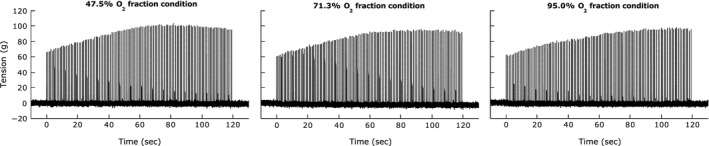
Representative muscle tension generation during muscle contraction for 120 sec under each O_2_ fraction conditions. Maximal twitch muscle contractions were elicited every 1 sec (1 Hz) by stimulating sciatic nerve. The time courses of the change in twitch tension at each O_2_ fraction condition are shown. No sign of fatigue was observed during muscle contraction regardless of the O_2_ fraction conditions.

Relative to the resting m$\dot{V}$O_2_, muscle contracting at a similar tension (77‐83 g) increased m$\dot{V}$O_2_ under all O_2_ supply conditions. The representative time course of the change in twitch tension at each O_2_ fraction condition was shown in Figure 1. However, the largest increase occurred at 95% O_2_, where the ∆m$\dot{V}$O_2_ rose by 0.28 *μ*mol g^−1^ min^−1^. At 47.5% and 71.3% O_2_ saturation, ∆m$\dot{V}$O_2_ rose only by 0.11 and 0.16 *μ*mol g^−1^ min^−1^, respectively. Even though m$\dot{V}$O_2_ increased under hypoxia conditions, it increased much less than muscle under normoxic condition (Table 2).

**Table 2:** Muscle oxygen consumption and intracellular O_2_ parameter during muscle contraction during hindlimb perfusion with different O_2_ fraction

Parameter	Unit	O_2_ fraction in perfusate		
		47.5%	71.3%	95.0%
Muscle tension	g	76.7 ± 15.2	81.5 ± 8.9	82.8 ± 12.9
m$\dot{V}$O_2_	*μ*mol g^−1^ min^−1^	0.45 ± 0.08^a^, †	0.60 ± 0.07^a^	0.87 ± 0.15
∆m$\dot{V}$O_2_	*μ*mol g^−1^ min^−1^	0.11 ± 0.04^a^, ^b^	0.16 ± 0.07^a^	0.28 ± 0.05
Outflow PO_2_	mmHg	104.9 ± 20.6^a^, †	158.7 ± 30.1^a^	252.2 ± 41.1
Relative outflow PO_2_		0.42 ± 0.08^a^, ^b^	0.63 ± 0.12^a^	1.00 ± 0.15
Estimated P_cap_O_2_	mmHg	12.5 ± 2.4^a^, ^b^	18.9 ± 3.6^a^	30.0 ± 4.9
S_mb_O_2_ kinetics				
Steady‐state value	%	12.3 ± 8.0^a^, ^b^	42.0 ± 16.6^a^	68.7 ± 3.0
AP	%	−42.7 ± 7.5^a^	−32.1 ± 8.8	−21.4 ± 3.0
MRT	s	39.4 ± 7.8	37.3 ± 8.0	42.4 ± 11.8
_0.63_AP/MRT	% s^−1^	−0.69 ± 0.10^a^	−0.57 ± 0.23^a^	−0.34 ± 0.11
P_mb_O_2_ kinetics				
Steady‐state Value	mmHg	0.4 ± 0.3^a^, ^b^	2.1 ± 1.2^a^	5.5 ± 0.9
AP	mmHg	−2.7 ± 0.8^a^	−6.5 ± 3.8^a^	−16.2 ± 0.9
MRT	sec	30.6 ± 5.4	35.3 ± 8.2	33.4 ± 11.7
_0.63_AP/MRT	mmHg sec^−1^	−0.03 ± 0.07^a^	−0.13 ± 0.18^a^	−0.41 ± 0.14
Intracellular [O_2_]	*μ*mol/L	0.5 ± 0.4^a^, ^b^	2.8 ± 1.7^a^	7.4 ± 1.2
P_cap_O_2_‐P_mb_O_2_	mmHg	12.1 ± 2.5^a^, ^a^	16.8 ± 3.8^a^	24.5 ± 2.5
∆L/P		3.6 ± 3.4^a^	2.5 ± 1.7	1.8 ± 1.0

Values are mean ± SD (*n* = 5 in each condition). m$\dot{V}$O_2_, muscle oxygen consumption; ∆m$\dot{V}$O_2_, the net increase in m$\dot{V}$O_2_ due to muscle contraction; Outflow PO_2_: oxygen tension after perfusion of the hindlimb, Relative outflow PO_2_, relative value of outflow PO_2_ based on the outflow PO_2_ value at 95.0 %O_2_ fraction; Estimated P_cap_O_2_, estimated microvasucular oxygen tension based on 30 mmHg of P_cap_O_2_ at 95.0% O_2_ fraction; S_mb_O_2_, intracellular O_2_ myoglobin saturation; AP is the amplitude between BL (baseline) and the steady‐state value during the exponential component; MRT is the time required to reach 63% of AP from the onset of muscle contraction. _0.63_AP/MRT is calculated by dividing _0.63_AP by MRT; P_mb_O_2_, intracelluar O_2_ tension equilibrated with myoglobin O_2_ saturation; Intracellular [O_2_], intracellular O_2_ concentration; P_cap_O_2_‐P_mb_O_2_, the difference in oxygen tension between P_cap_O_2_‐P_mb_O_2_; ∆L/P, the net increase in lactate to pyruvate ratio measured in effluent perfusate.

a *P* < 0.05 versus 95.0% condition.

b *P* < 0.05 versus 71.3% condition.

In contrast, L/P increased with declining O_2_ supply. L/P increased significantly higher at 47.5% O_2_ (3.6 ± 3.4) than at 95.0% O_2_ (1.8 ± 1.0). At 95.0% O_2_ the muscle contraction causes no significant change in the L/P ratio, consistent with previous observations (Takakura et al. 2010).

**Figure 2:**
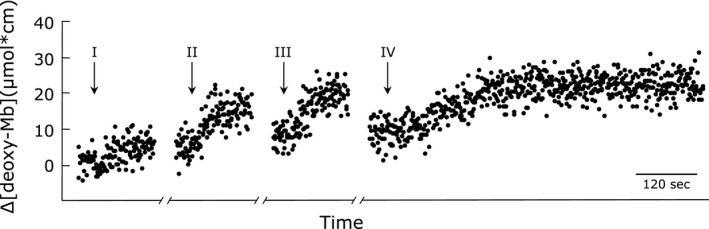
Representative time courses of the ∆[deoxy‐Mb] NIRS signals during muscle contraction under different O_2_ fraction conditions (I–III) and during anoxia perfusion (IV). The arrows indicate the onset of muscle contraction (I–III) and anoxic perfusion (95%N_2_ + 5%CO_2_; IV). Protocols I, II and III show the ∆[deoxy‐Mb] signals during maximal twitch contraction under 95.0%, 71.3% and 47.5% O_2_ fraction conditions in the Hb‐free perfusion model. The ∆[deoxy‐Mb] signals changed immediately at the onset of contraction and reached the steady state. As protocol IV was conducted after finishing protocol III, desaturated Mb already existed at a certain level at rest before perfusing anoxia buffer.

During muscle contraction, the steady S_mb_O_2_ and P_mb_O_2_ still decreased with decreasing oxygen supply. However, the S_mb_O_2_ and P_mb_O_2_ kinetics did not show a significant difference (Table 2). Figure 2 shows representative ∆[deoxy‐Mb] kinetics during different levels of O_2_ delivery conditions and during anoxic perfusion, as assessed by NIRS. The roman numerals I, II and III represent the NIRS signal response to 1 Hz maximal twitch contractions at 95.0%, 71.3% and 47.5% O_2_ fraction condition, respectively. In protocol IV, the noncontracting muscle received a perfusate equilibrated with 95% N_2_ + 5% CO_2_.

**Figure 3:**
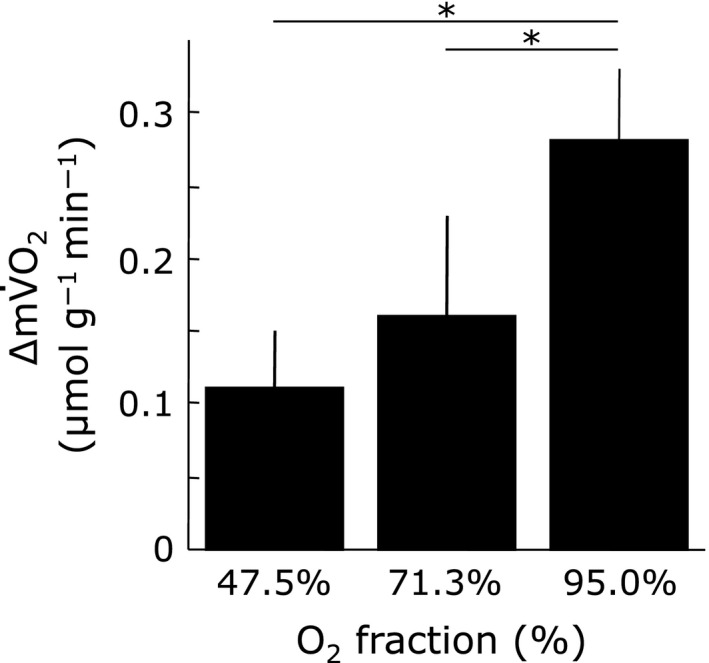
Change in net increase in muscle O_2_ consumption (∆m$\dot{V}$O_2_) due to muscle contraction for each O_2_ fraction. The ∆m$\dot{V}$O_2_ due to muscle contraction decreased with the decrease in the O_2_ supply volume. ∆m$\dot{V}$O_2_: net increase in muscle oxygen consumption due to contraction. Values are expressed as means ± SD (*n* = 5). *P < 0.05 vs. 95.0% O_2_ fraction.

**Figure 4:**
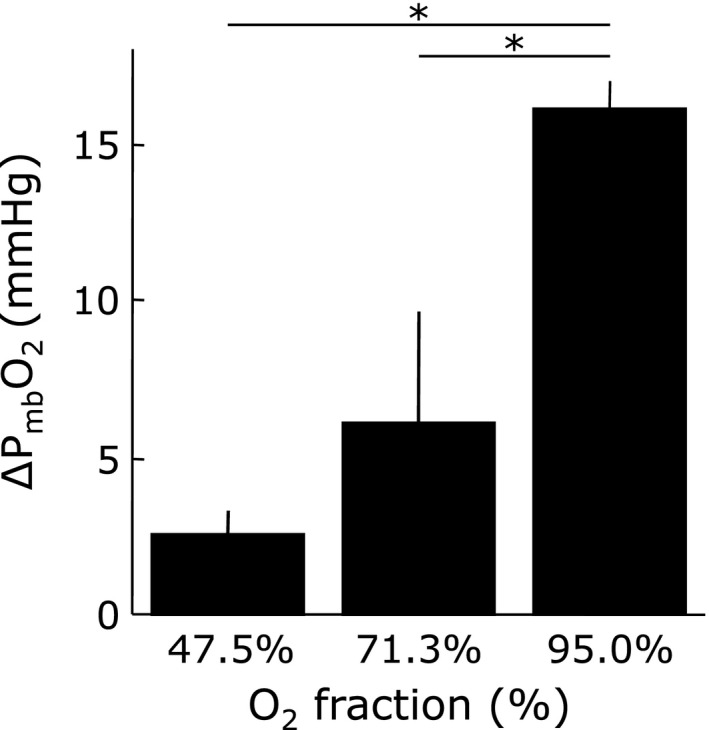
Net decrease in intracellular oxygen tension (∆P_mb_O_2_) due to muscle contraction for each O_2_ fraction. The ∆m$\dot{V}$O_2_ due to muscle contraction decreased with the decrease in the O_2_ supply volume. P_mb_O_2_ during contraction, hatched bars: net decrease in P_mb_O_2_. ∆P_mb_O_2_: net decrease in intracellular oxygen tension equilibrated with O_2_ saturation myoglobin. Values are expressed as means ± SD (*n* = 5). *P < 0.05 vs. 95.0% O_2_ fraction.

**Figure 5:**
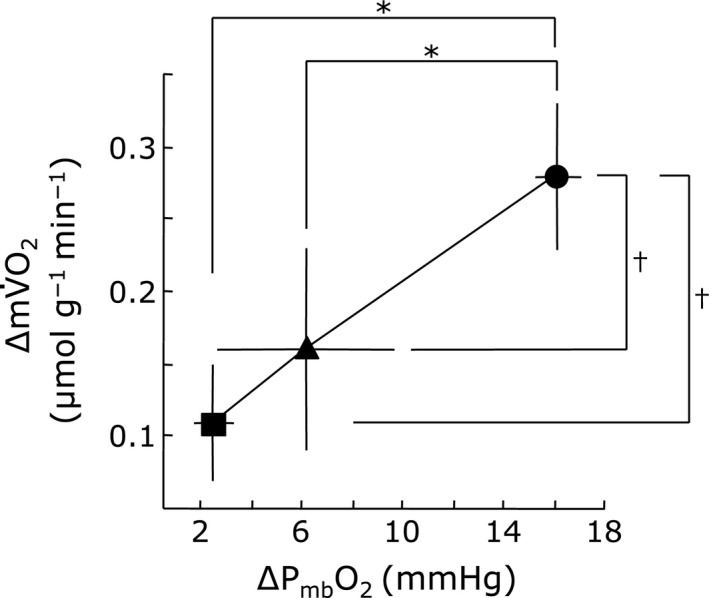
Relationship between net decrease in intracellular oxygen tension (∆P_mb_O_2_) and net increase in muscle oxygen consumption (∆m$\dot{V}$O_2_) during muscle contraction. The ∆P_mb_O_2_ and ∆m$\dot{V}$O_2_ gradually decreased as the O_2_ supply volume decreased. The relationship between the ∆P_mb_O_2_ and the ∆m$\dot{V}$O_2_ was represented with a line graph. ∆m$\dot{V}$O_2_: net increase in muscle oxygen consumption due to contraction. ∆P_mb_O_2_: net decrease in intracellular oxygen tension equilibrated with O_2_ saturation myoglobin. Each data point represents a mean ± SD. *n* = 5 × 3 points. *P < 0.05 for ∆m$\dot{V}$O_2_ parameter. ^†^ *P* < 0.05 for ∆P_mb_O_2_ parameter.

Figure 3 shows the net increase in m$\dot{V}$O_2_ (∆m$\dot{V}$O_2_) relative to its resting value during muscle contraction in each O_2_ fraction condition. The ∆m$\dot{V}$O_2_ due to muscle contraction (0.11 ± 0.04, 0.16 ± 0.07, and 0.28 ± 0.05 *μ*mol g^−1^ min^−1^ at 47.5%, 71.3%, and 95% O_2_ fraction) decreased progressively with hypoxia. Under resting conditions, the measurements indicate P_mb_O_2_ values of 3.1, 8.5, and 21.7 mmHg at 47.5, 71.3, and 95% O_2_. With muscle contraction, the P_mb_O_2_ values decrease correspondingly to 0.4, 2.1, and 5.5 mmHg. Figure 4 shows then net decrease in P_mb_O_2_ (∆P_mb_O_2_) due to muscle contraction (2.50 ± 0.81, 6.12 ± 3.52, and 16.10 ± 0.87 mmHg in 47.5, 71.3, and 95% O_2_ fraction conditions, respectively). Even though P_mb_O_2_ decreases under all exercise conditions, the value changes the most at 95% O_2_. Figure 5 shows the relationship between the ∆m$\dot{V}$O_2_ and the ∆P_mb_O_2_ in each O_2_ fraction condition. During muscle contraction, the decrease in oxygen supply caused a decrease in the ∆P_mb_O_2_, which led to a decrease in the ∆m$\dot{V}$O_2_.

**Figure 6:**
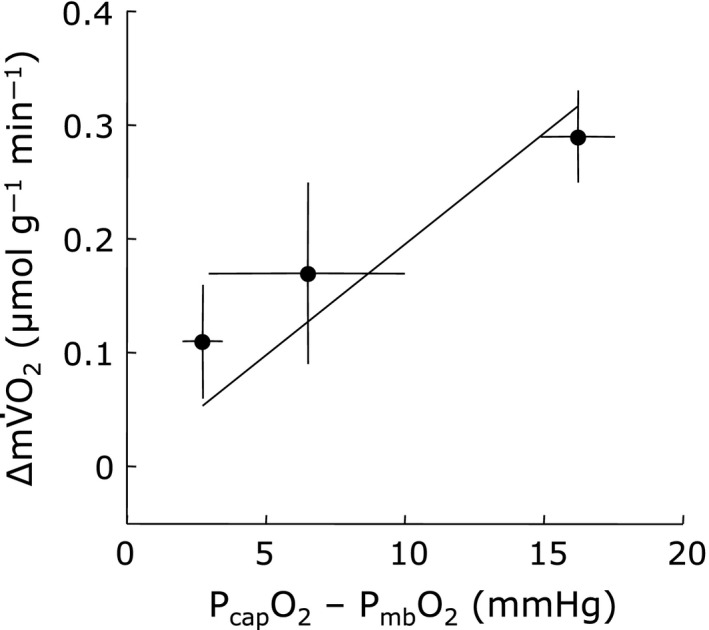
Relationship between delta change in muscle oxygen consumption (∆m$\dot{V}$O_2_) and estimated O_2_ gradient between microvascular oxygen tension (P_cap_O_2_) and intracellular oxygen tension (P_mb_O_2_) under exercising condition. Delta change in m$\dot{V}$O_2_ due to muscle contraction increased linearly as a function of estimated O_2_ gradient (P_cap_O_2_‐P_mb_O_2_). Regression line is based on mean values (m$\dot{V}$O_2_ = 0.020 × (P_cap_O_2_‐P_mb_O_2_), *R* ^*2*^ = 0.99, *n* = 5 in each point). Each data point represents a mean ± SD.

The linear relationship between ∆m$\dot{V}$O_2_ and ∆P_mb_O_2_ suggests that the O_2_ gradient as reflected in (P_cap_O_2_‐P_mb_O_2_) has changed. Using an estimate P_Cap_O_2_, the analysis shows that indeed ∆m$\dot{V}$O_2_ changes linearly with (P_cap_O_2_‐P_mb_O_2_) (Fig. 6).

## Discussion

### Intracellular O_2_ environment under hypoxic conditions

As oxygen delivery decreases, the P_mb_O_2_ at rest decreased correspondingly from 21.7 to 8.5 to 3.1 mmHg. The m$\dot{V}$O_2_ decreases correspondingly from 0.55 to 0.43 to 0.34 *μ*mol g^−1^ min^−1^. It might appear that the decreasing O_2_ supply leads consequently and directly to the decreasing m$\dot{V}$O_2_. Yet in normoxic muscle the decreasing intracellular O_2_ leads to an increasing m$\dot{V}$O_2_, which implies a critical role for the O_2_ gradient instead of the O_2_ supply alone. An analysis of the O_2_ gradient as reflected in (P_cap_O_2_‐P_mb_O_2_) shows that despite the decreasing intracellular O_2_, the O_2_ gradient appears to have increased during hypoxia. The low m$\dot{V}$O_2_ in resting muscle most likely creates a shallow gradient, which obscures measurement accuracy. Nevertheless, the constant L/P ratio would argue that the O_2_ supply and m$\dot{V}$O_2_ have not compromised oxidative metabolism, which would require glycolysis to compensate for any energy deficit. Consistent with a previous findings, the decreasing O_2_ supply did not affect the low m$\dot{V}$O_2_ at rest (Shiota and Sugano 1986).

However, when muscle begins to contract, m$\dot{V}$O_2_ rose and the P_mb_O_2_ decreased further from the resting levels. At 47.5% O_2_, the P_mb_O_2_ drops below the critical PO_2_ level that limits oxidative phosphorylation in mitochondria (1.5 mmHg) (Kreutzer et al. 1992). L/P rises more at 47.5% O_2_ than at 95.0% O_2_. However, the drop in P_mb_O_2_ also expands the O_2_ gradient under all O_2_ saturation conditions during muscle contraction. But the widening O_2_ gradient does not sufficiently enhance the O_2_ flux to meet the cell's oxidative needs. Anaerobic glycolysis commences to supplement the energy demand, especially as the buffer PO_2_ drops to 47%.

These findings indicate that even under hypoxia m$\dot{V}$O_2_ rises during contraction. Given the same O_2_ saturation conditions, the rise in m$\dot{V}$O_2_ required an increase in DO_2_ or (P_cap_O_2_‐P_mb_O_2_) as shown in equation (Richardson et al. 1995; Takakura et al. 2010). Even though the P_mb_O_2_ decreases, the O_2_ gradient has widened to increase the O_2_ flux to balance out the O_2_ supply and demand. A decreasing P_mb_O_2_ with a rising m$\dot{V}$O_2_ agree with the previously reported observations (Molé et al. 1999; Takakura et al. 2010). In contrast, Richardson et al. (1995) reported that under normoxic or hypoxic condition, the P_mb_O_2_ did not decrease proportionally to muscle contraction, once the contraction has exceeded about 40% of maximum work exercise workload. Even under hypoxic condition, the Mb desaturation level does increase as the leg $\dot{V}$O_2_ rose to its maximum level (Richardson et al. 1995). In addition, the P_mb_O_2_ reaches a low level (<5 mmHg) in the skeletal muscle, even when moderate levels of work were performed (Molé et al. 1999; Chung et al. 2005; Takakura et al. 2010). These findings have led researchers to ignore the P_mb_O_2_ variable from the formula for the calculation of the amount of O_2_ consumption in the muscle tissue in hypoxic environments (Gonzalez et al. 2006).

However, probably due to a signal‐to‐noise ratio limitation, Richardson et al. (2006) could not detect the P_mb_O_2_ at rest in hypoxic muscle. As a result, the study could not determine the true magnitude of the change in the intracellular oxygen environment during exercise. Our study's unique experimental model, with the subjects placed under Hb‐free perfusion, suggests that changes in the intracellular oxygen environment and the O_2_ gradient can contribute to the Δm$\dot{V}$O_2_ during hypoxia. In addition, the P_mb_O_2_ at the resting muscle did not reach a nadir as a result of the hypoxia. Instead, the P_mb_O_2_ can decreased further during the muscle contraction even in the hypoxic conditions.

### Change in P_mb_O_2_ from muscle contraction modulating change in m$\dot{V}$O_2_ under Hb‐Free perfusion

The study investigated whether the m$\dot{V}$O_2_ during muscle contraction in various oxygen concentration conditions depend on the changes in the P_mb_O_2_ or the O_2_ gradient. Because the oxygen delivery remained constant at any muscle contraction condition in the perfusion model at a constant flow, the decrease in P_mb_O_2_ due to muscle contraction widen the O_2_ gradient between the microvasculature and myocyte as reported in the our previous study (Takakura et al. 2010). This change contributes the increase in m$\dot{V}$O_2_ due to muscle contraction. This interpretation is proven by the following equation;

$$m\dot{V}O_{2}={\text{kDO}}_{2}\times {\text{(P}}_{\mathrm{cap}}O_{2}-P_{\mathrm{mb}}O_{2})$$

Because kDO_2_ does not significantly contribute to the increase in m$\dot{V}$O_2_ due to muscle contraction in the constant‐flow hindlimb perfusion model, the m$\dot{V}$O_2_ cannot increase unless the O_2_ gradient expands by decrease in P_mb_O_2_ in our previous studies (Takakura et al. 2010, 2015). In the present study, the results have confirmed the hypothesis that the delta change in P_mb_O_2_ and in the O_2_ gradient contributes to delta increase in m$\dot{V}$O_2_ during muscle contraction. Our results show that under all oxygenation conditions, m$\dot{V}$O_2_ rises but P_mb_O_2_ continues to decline by muscle contraction between 77% and 88% of maximal voluntary contraction (MVC). However, rise in the m$\dot{V}$O_2_ and O_2_ gradient expansion under hypoxia conditions are much less than the corresponding changes under normoxic conditions. The observation then agree with the studies by Molé et al. (1999) and Takakura et al. (2010), which indicate the progressive expansion of the O_2_ gradient between the P_cap_O_2_ and the P_mb_O_2_ during muscle contraction contribute to the O_2_ flux supporting the rising m$\dot{V}$O_2_. No P_mb_O_2_ plateau even under hypoxia condition seems apparent at MVC well above 40%.

Moreover, a changing m$\dot{V}$O_2_ in constant flow model also supports a P_mb_O_2_ contribution. In constant flow perfusion, a constant flow‐induced vasodilatation occurs at a given flow rate. As a consequence, the model maintains a constant diffusion conductance throughout the perfusion (Hepple et al. 2003). Consequently, any change in ∆m$\dot{V}$O_2_ must have a contribution from P_mb_O_2_. Therefore, the mechanism behind the m$\dot{V}$O_2_ in hypoxic environments, includes then a contribution P_mb_O_2_ (Molé et al. 1999; Takakura et al. 2010).

The mechanism of the oxygen transport to the mitochondria in the myocytes is dependent on the Mb‐mediated oxygen transport or the dissolved O_2_ flux. The contribution from the Mb‐mediated oxygen transport increases with decreasing P_mb_O_2_. Below the equipoise diffusion PO_2_ value (1.77 mmHg), 50% of the total oxygen transport to the mitochondria stems from the Mb‐mediated oxygen transport (Lin et al. 2007). The P_mb_O_2_ during muscle contraction in the 47.5% O_2_ fraction condition was 0.4 ± 0.3 mmHg. As this was below the equipoise diffusion PO_2_ value, the Mb‐mediated oxygen transport mechanism will dominate. In addition, any increase in Mb concentration will raise the equipoise PO_2_ (Lin et al. 2007; Takakura et al. 2015). Because people acclimated to high altitudes often have a high concentration in Mb, muscle contraction under hypoxic conditions in these people may also benefit from Mb‐mediated transport (Reynafarje 1962; Reynafarje and Morrison 1962).

Van der Laarse et al. (2005) have examined about the value of the diffusion coefficient for oxygen from extracellular region in muscle. The diffusion coefficient is important because it is a determinant of the extracellular oxygen tension at which the core of muscle fibers becomes anoxic (PO_2crit_). According to Hill (1965), PO_2crit_ at the maximum rate of oxygen consumption ($\dot{V}$O_2max_; in nmol mm^−3^ sec^−1^) is given by

$${\text{PO}}_{2}\text{crit}=\dot{V}O_{2}\text{max}\times \text{CSA}/4\pi D\alpha$$

where CSA is the cross‐sectional area of the cell (mm^2^), D is the diffusion coefficient for oxygen in the muscle cell (mm^2^/sec), and *α* is the solubility of oxygen in muscle cell (mmol/L per mmHg). D*α* is known as Krogh's diffusion coefficient. D*α* seems to depend on fat content, extracellular space, and unknown factors (Jones and Kennedy 1986; Dutta and Popel 1995; Baranov et al. 2000). Indeed, D*α* increased with enlargement of extracellular space in the preparation (van der Laarse et al. 2005). However, the results of the previous study indicated that Hill's model for oxygen diffusion in valid for single muscle fibers without myoglobin and a fairly homogeneous mitochondrial distribution consuming oxygen at the maximum rate. Because above Hill's equation is based on the following assumption: (1) the cross section of the fiber is a circle, and oxygen diffuses in the radial direction only; (2) $\dot{V}$O_2_ is distributed homogeneously in the cells; (3) $\dot{V}$O_2_ by mitochondria is independent of local intracellular PO_2_; and (4) myoglobin‐facilitated oxygen diffusion is negligible, the role of Mb was not taken account of above D*α*. We have conducted the maximal twitch contraction under three O_2_ fraction conditions to the same individual in the present perfusion model. Moreover, as the order of the three O_2_ condition was randomized and no sign of edema in the hindlimb was seen at the given flow rate, change in extracellular space would be almost none. Thus, D*α* in the above equation would not been affected throughout the perfusion experiment in this study. On the other hand, others and we reported the effect of Mb on the intracellular O_2_ diffusion to the mitochondria (Lin et al. 2007; Takakura et al. 2010, 2015). As mentioned above, Lin et al. (2007) reported that 50% of the total oxygen transport to the mitochondria stems from the Mb‐mediated oxygen transport below the equipoise diffusion PO_2_ value (1.77 mmHg). Also, we reported that decrease in P_mb_O_2_ directly contribute the expansion of the O_2_ gradient to lead the increased m$\dot{V}$O_2_ (Takakura et al. 2010, 2015). As for a key factor regulating O_2_ diffusion to intracellular mitochondria, the change in P_mb_O_2_ might be one of candidates, because the change in P_mb_O_2_ affects O_2_ gradient, Mb‐mediated O_2_ transport and mitochondria respiratory.

## Conclusion

This study investigated the effects of reduced oxygen supply on the P_mb_O_2_ at rest and during muscle contraction, as well as the effect of the ΔP_mb_O_2_ on the Δm$\dot{V}$O_2_ during muscle contraction. Our findings showed that the resting P_mb_O_2_ decreases with the decrease in oxygen supply. However, the decrease in P_mb_O_2_ expands the O_2_ gradient, which then supports the rising ∆m$\dot{V}$O_2_ during muscle contraction even under hypoxic conditions. The results suggest that the ΔP_mb_O_2_ is a key determinant factor of the Δm$\dot{V}$O_2_.

## Conflict of interest

There is no conflict of interest for this study.
